# Weak Te, Te Interactions through the Looking Glass of NMR Spin–Spin Coupling**

**DOI:** 10.1002/anie.201205998

**Published:** 2013-01-23

**Authors:** Michael Bühl, Fergus R Knight, Anezka Křístková, Irina Malkin Ondík, Olga L Malkina, Rebecca A M Randall, Alexandra M Z Slawin, J Derek Woollins

**Affiliations:** EaStCHEM School of Chemistry, University of St. AndrewsNorth Haugh, St. Andrews, Fife KY16 9ST (UK); Slovak Academy of Sciences, Institute of Inorganic Chemistry84536 Bratislava (Slovakia); Faculty of Mathematics, Physics and Informatics, Comenius UniversityBratislava, 842 48 Bratislava (Slovakia); * EaStCHEM School of Chemistry, University of St. AndrewsNorth Haugh, St. Andrews, Fife KY16 9ST (UK)

**Keywords:** naphthalenes, quantum-chemical computation, tellurium, through-bond interactions, through-space interactions

Most of the tools for quantifying the extent of chemical bonding between two atoms are quantum-chemical in nature. None of them are unambiguous, however, and different analyses can lead to conflicting interpretations, even concerning the most fundamental question of whether or not atoms are linked by a chemical bond.^1^, ^2^ One of the indicators that can be probed experimentally is the indirect spin–spin coupling constant (SSCC). For instance, observation of spin–spin coupling across hydrogen bonds^3^ has been taken as evidence for covalent contributions to this kind of bonding. For atoms within the same molecule that are close in space, but not linked through a direct formal bond, the question arises, how much of the observed coupling is transmitted through a succession of bonds that eventually links them (“through-bond coupling”), and how much is due to interaction through the overlap of lone pairs (“through-space coupling”). Again, a variety of quantum-chemical tools have been developed to address this question.

A rigid scaffold that is used to achieve such spatial proximity is the naphthalene framework, in which substituents in the *peri* (1,8) positions have a typical separation of around 3 Å.^4^ Through-space *J*(^19^F,^19^F) SSCCs, long known for their distance dependence,5a have been studied in some detail in *peri*-difluoronaphthalenes.5b,c Similarly, *J*(^31^P,^31^P) values in *peri*-bis(phosphino)naphthalenes have been attributed to through-space coupling,^6^ and *J*(^77^Se,^77^Se) values in *peri*-bis(seleno) derivatives have been analyzed in detail through quantum-chemical computations.^7^ In systematic studies of naphthalene (**N**) and acenaphthene (**A**) derivatives with pnictogen and chalcogen atoms in the *peri* positions, it became apparent that for the heavier congeners, steric repulsion is partly counterbalanced by attractive interactions. In particular with Te substituents, formally nonbonded, “across-the-bay” distances are significantly shorter than the sum of the van-der-Waals radii, which has been traced back to weak donor–acceptor interactions and the onset of 3-center-4-electron (3c4e) bonding.^8^

For instance in **N1** and **A1** (Scheme 1), Te⋅⋅⋅Te distances of around 3.3 Å are observed in the solid state (ca. 0.7 Å below the sum of the vdW radii), and Wiberg bond indices^9^ (WBIs, a measure for the covalent character of a bond, approaching WBI=1 for a true single bond) of around 0.15 have been computed. Slightly larger WBIs of approximately 0.18 have been obtained for cationic methylated species **N2** and **A2**, despite a slightly longer Te–Te separation (ca. 3.4 Å).^10^ These unsymmetrical systems show remarkably large *J*(^125^Te,^125^Te) SSCCs, formally ^4^*J* values, of 1093.0 Hz and 945.8 Hz in **N2** and **A2**, respectively.^10^ We now report even larger couplings in **N1** and **A1**, along with computational conformational analysis underlining the potential of this property as a structural and interpretative method.

**Scheme 1:**
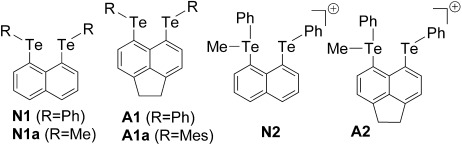
*Peri*-napthalene ditellurides with formally nonbonded Te atoms.

The computed (ZORA-SO/BP//B3LYP level)^11^ *J*(^125^Te,^125^Te) SSCCs in **N2** and **A2** (1490 and 1377 Hz, respectively) are noticeably overestimated with respect to the experiment, but are in the right order of magnitude and reproduce the relative tendency rather well. At the same level, *J* values of 2779 and 1543 Hz were predicted for **N1** and **A1**, respectively, showing a larger discrimination between the two types of compounds. As will be shown below, this discrimination is largely due to subtle conformational effects.

Because the two Te sites in **N1** and **A1** are magnetically equivalent in solution, direct observation of *J*(^125^Te,^125^Te) is not possible, but the coupling can be detected as satellites in the ^123^Te NMR spectrum (see experimental details). With this technique, *J*(^123^Te,^125^Te) values of 2077 and 1750 Hz are obtained for **N1** and **A1**, respectively. Neglecting primary and secondary isotope effects, these values correspond to 2505 and 2110 Hz, respectively, in *J*(^125^Te,^125^Te). While the computed value for **N1** is again overestimated with respect to the experiment, that for **A1** is underestimated, but overall, the predicted tendency is confirmed qualitatively.

To gain deeper insights into the factors that govern the magnitude of these *J* values, additional computations were performed (Table 1). Typical for NMR properties of heavier elements,^12^ the theoretical *J* couplings are quite sensitive to relativity (compare entries 1–3 in Table 1), level of geometry optimization (compare entries 3 and 4), exchange–correlation functional (compare entries 4 and 5), and rather strongly on the conformation (compare entries 7 and 8).

**Table 1:** Computed *J*(^125^Te,^125^Te) couplings in *peri*-naphthalene ditelluride derivatives.

Entry	Compound	Level of theory^[a]^	*J*_FC_^[b]^ [Hz]	*J*_PSO_^[c]^ [Hz]	*J*_tot_^[d]^ [Hz]
1	**N1**	NR/BP//B3LYP	1872	−2	1870
2	**N1**	ZORA/BP//B3LYP	2874	−11	2863
3	**N1**	ZSO/BP//B3LYP	2858	−79	2779
4	**N1**	ZSO/BP//PBE0	3373	−38	3335
5	**N1**	ZSO/PBE0//PBE0	3707	−46	3661
6	**N1 a**	ZSO/BP//B3LYP	2739	−48	2691
7	**A1**	ZSO/BP//B3LYP	2640	−36	2604
8	**A1**^[e]^	ZSO/BP//B3LYP	1713	−170	1543

[a] Notation “level of NMR computation//level of geometry optimization” (CCt conformer except where otherwise noted); NR: nonrelativistic, ZSO: Zora-spin-orbit, TZ2P basis employed throughout. [b] Sum of Fermi-contact and spin-dipolar part. [c] Paramagnetic spin-orbit part. [d] Total *J* coupling. [e] AB conformer.

The conformations of each phenyl group with respect to the naphthalene plane can be classified as roughly perpendicular (A), in-plane (B), or in-between (C), and both can be *cis* (c) or *trans* (t) to each other.^8^ In the solid, **N1** and **A1** adopt CCt and AB conformations, respectively (see Figure S1 in the Supporting Information). For the smaller methyl derivative **N1 a**, for which a *J* value very similar to that of **N1** is computed (compare entries 3 and 6 in Table 1), a detailed conformational analysis was carried out. Results as a function of the two C^9^-C-Te-C(Me) dihedral angles are summarized in two-dimensional (2D) Ramachandran-type plots (Figure 1).

**Figure 1:**
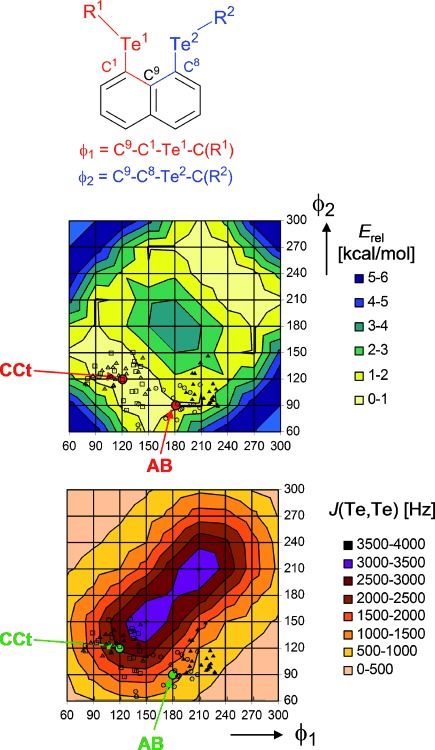
Relative energies (middle, B3LYP level) and (^125^Te,^125^Te) coupling constants (bottom, ZORA-SO/BP level) in **N1 a** as a function of the conformation, as defined by the two dihedral angles specified at the top (R^1^=R^2^=Me, all other parameters B3LYP-optimized). Open and filled black symbols: conformations visited during a molecular dynamic simulation (see main text).

The first of the 2D plots represents the potential energy, with the minimum labeled as CCt. There is a huge flat area within just 1 kcal mol^−1^ (light yellow) which extends well into what would be classified as AB conformation, consistent with the structural variety observed in the solid for different members of this family.^8^ The second of the 2D plots represents the *J* coupling surface, which looks quite different. Here, a structure with CCt conformation would have a *J* value of around 2500 Hz, similar to what is found for **N1**; a structure in the AB region would be much lower (ca. 1500 Hz). This situation is reminiscent of that in the Se congeners, although the *J*(^77^Se,^77^Se) values computed for the corresponding stationary points are much smaller.^7^, ^13^

From the flatness of the potential energy surface, it can be anticipated that the systems will sample large areas in the conformational space around the minima. This expectation is borne out in a short molecular dynamics (MD) simulation of **N1 a** at the B3LYP level, in the course of which a large territory on the 2D map is visited. The black symbols in Figure 1 are snapshots taken every 100 fs between 2–10 ps of total MD simulation starting from the CCt minimum, demonstrating that not only the vicinity of this minimum is sampled, but also slightly higher-lying areas around an AB conformation. The mean *J* coupling computed over all these snapshots is reduced significantly, by around 900 Hz, from the equilibrium value in Table 1 (ca. 2700 Hz). While this thermal correction would need much longer simulation times for full convergence (see Figure S2), it is clear that single, static NMR computations will not be sufficient to reach a quantitative agreement with the experiment.^14^ Taken together, however, the computational data enable a consistent interpretation of the observations: In solution, **N1** and **A1** are rather fluxional, and the observed variation in the *J*(^125^Te,^125^Te) SSCCs are indicated to arise from subtle shifts in the populations of few conformers (or rather, larger areas in conformational phase space around them). Because the *J* coupling changes so dramatically with the conformations (bottom of Figure 1), it is a sensitive probe into structure and dynamics of this kind of system.

A mean *J*(^125^Te,^125^Te) value of more than 2500 Hz, as observed for **N1**, is quite large for a ^4^*J* coupling between formally nonbonded Te atoms. The largest SSCC between bonded Te atoms is, to our knowledge, ^1^*J*(^125^Te,^125^Te)=4395 Hz in Te_2_^2−^.^15^ Similar values can be reached in **N1 a** (Figure 1) and are even exceeded in one of the instantaneous MD snapshots. Why are these formal ^4^*J* couplings so large? When the naphthalene moiety in the B3LYP geometry of **N1** is deleted and the Te atoms capped with H atoms, the computed *J* value even increases from 2779 Hz to 3101 Hz. Transmission through the intermittent TeC- and CC-bonds thus plays only a minor role.

The same conclusion can be drawn from analysis of the Te–Te coupling pathway in **N1 a** through inspection of the coupling deformation density (CDD):^16^ Reminiscent of the situation in ^*n*^J(P,P) couplings (*n*=2,3),^16^ the overlap of the lone pairs (lps) is the determining mechanism, rather than transmission through the organic framework (Figure 2). The CDD topology was obtained for the first time at a relativistic level (the first order Douglas-Kroll-Hess, DKH-1). Because it is very similar to its nonrelativistic counterpart (see Figure S3 in the Supporting Information), the analysis of *J*(^125^Te,^125^Te) performed at the nonrelativistic level should give a qualitatively correct picture. At the nonrelativistic BP level, the total calculated FC part of *J*(^125^Te,^125^Te) is 2038 Hz, the largest part of which (in a localized MO formalism with Pipek-Mezey localization^17^) stems from the two “sp-like”^18^ Te lone pairs (+2359 Hz in total), with smaller contributions from the four Te–C bonds (−301 Hz) and negligible ones from the two “p-type” Te lone pairs (21 Hz, see Figure S4 in the Supporting Information).

**Figure 2:**
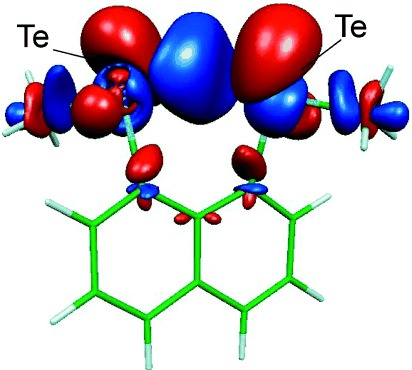
Total coupling deformation density (CDD) in **N1 a**. (DKH-1/BP/Hirao/II level, isosurfaces plotted for CDD=6.0).

It is noteworthy that the Te,Te coupling path in the CDD analysis extends noticeably into the “dead ends” of the Te–C(Me) bonds, rather than the conjugated aromatic moiety. This may be taken as evidence for the importance of the lp(Te)→σ*(TeC) interaction that is responsible for the onset of 3c4e bonding and the noticeable WBI.^8^, ^19^ In this context, it is interesting to note that at the same nonrelativistic level, a bond-critical point is located between the two Te atoms in **N1 a** in an atoms-in-molecules analysis.^20^

The variation in the computed *J* values can be rationalized through the extent of overlap between the densities of the Te lone pairs (Figure S5). Compared to typical “through-space” ^*n*^*J*(^31^P,^31^P) couplings, the Te,Te couplings of this study are larger by at least one order of magnitude.^13^ From computed valence s-orbital densities (see the Supporting Information) and the gyromagnetic ratios, one would expect Te,Te couplings to be approximately seven times larger than the corresponding *J*(P,P). Further enhancement is likely due to the increased polarizability and the onset of multicenter bonding in the Te systems.

In the course of our studies, we noticed that the mesityl derivative **A1 a** showed an even larger Te,Te coupling than **N1** or **A1**, namely *J*(^125^Te,^125^Te)=3398 Hz. This value led us to suspect that, unlike **A1** with its AB conformation,^8^ **A1 a** would rather adopt a CCt conformation, an expectation that was borne out in a subsequent structure determination by X-ray crystallography (Figure 3).^21^ These findings underscore the potential of Te,Te-coupling constants as an analytical tool.

**Figure 3:**
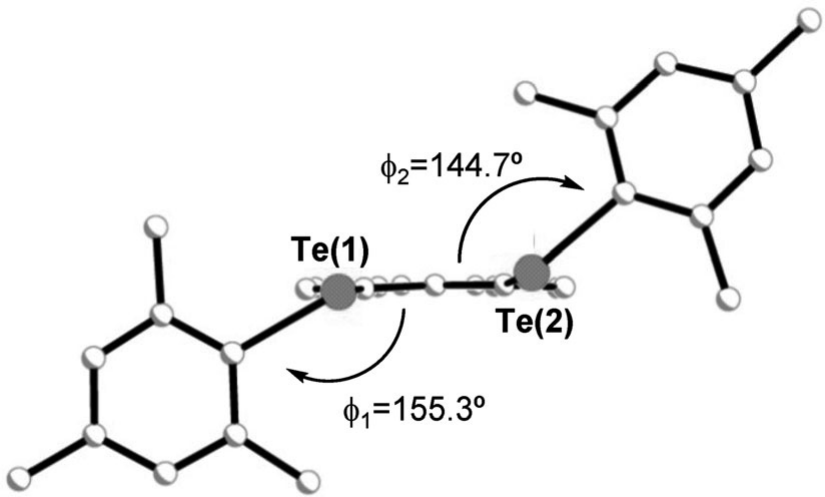
The crystal structure of 5,6-bis(mesityltelluro)acenaphthene (**A1 a**, H atoms omitted for clarity), including the key dihedral angles for classification as CCt conformation. See the Supporting Information for further details.

In summary, a combination of NMR and DFT techniques has been used to study the interactions between formally nonbonded, but spatially close Te atoms. Weak donor–acceptor interactions in the *peri*-naphthalene system, which mark the onset of 3c4e bonding, reinforce the Te,Te couplings and lead to unusually large *J*(^125^Te,^125^Te) values. This property turns out to be a sensitive probe (“looking glass”), not only into the electronic structure underlying the bonding situation, but also into the particular conformations that ensue. In the broader context of well-known through-space spin–spin coupling, this conformational aspect is a new facet worth exploring. Studies are under way to probe how substituents at the phenyl rings can modulate the bonding and the concomitant NMR properties, ever broadening our knowledge of the very foundation of chemistry, the chemical bond.

## Experimental Section

Geometry optimizations and Born–Oppenheimer MD simulations were carried out at the B3LYP/6-31G* level (augmented SDD on Te), *J* values computed^22^ at the ZORA-Spinorbit/BP86/TZ2P level (which has performed well for the computation of SSCCs involving fourth-row and heavier elements)^23^ or at the nonrelativistic BP86 level (TZVP on Te, IGLO-II on C,H).^11^ ^123^Te NMR spectra were recorded on a Jeol GSX 270 MHz spectrometer with *δ*(Te) referenced to external diphenyl ditelluride. ^123^Te NMR (70.7 MHz, 25 °C, PhTeTePh): **N1** (CDCl_3_): *δ*=620.3 ppm (s, ^4^*J*(^123^Te,^125^Te)=2077.4 Hz). **A1** (CDCl_3_): *δ*=586.6 ppm (s, ^4^*J*(^123^Te,^125^Te)=1750.4 Hz; see Figure S8 in the Supporting Information for the spectra), **A1 a** (CDCl_3_): *δ*=362.9 ppm (s, *J*(^123^Te-^125^Te) 2818.5 Hz; see Supporting Information for experimental details).

## Supplementary material

As a service to our authors and readers, this journal provides supporting information supplied by the authors. Such materials are peer reviewed and may be re-organized for online delivery, but are not copy-edited or typeset. Technical support issues arising from supporting information (other than missing files) should be addressed to the authors.
