# Supplementary material for: Weak Te, Te Interactions through the Looking Glass of NMR Spin–Spin Coupling
Source: Angew Chem Int Ed Engl. 2013 Jan 23;52(9):2495–8. doi: 10.1002/anie.201205998 (PMC3625736; doi:10.1002/anie.201205998)
Supplement: Supplementary file 1 [file anie0052-2495-SD1.pdf]

Angewandte Chemie
Eine Zeitschrift der Gesellschaft Deutscher Chemiker

Supporting Information

© Wiley-VCH 2013

69451 Weinheim, Germany

Weak Te,Te Interactions Through the Looking Glass of NMR Spin–Spin Coupling**

Michael Bühl, Fergus R. Knight, Anezka Křístková, Irina Malkin Ondík, Olga L. Malkina, Rebecca A. M. Randall, Alexandra M. Z. Slawin, and J. Derek Woollins*

anie_201205998_sm_miscellaneous_information.pdf

Full Computational Details

Geometries were fully optimized in the gas phase at the B3LYP level[1] using the Stuttgart-Dresden (SDD) effective core potential along with its double zeta valence basis sets for Te[2] (augmented with a set of d-polarization functions with exponent 0.237)[3] and 6-31G(d) basis elsewhere (see Figure S1 for 3D plots showing the lowest conformations). Wiberg bond indices[4] were obtained in a natural bond orbital analysis[5] at the same level. This or similar levels have been useful for interpreting experimental findings for *peri*-naphtalene telluride derivatives.[6] Selected structures were reoptimized using the PBE0 hybrid functional,[7] and nonrelativistic all-electron single-point calculations were performed for B3LYP/SDD geometries at the BP86 level[8] using TZVP basis from the Turbomole[9] library on Te (TZVPall, d-exponent 0.237) and IGLO-basis II[10] elsewhere. A fine integration grid (75 radial shells with 302 angular points per shell) was used throughout. These computations were performed using the Gaussian suite of programs.[11]

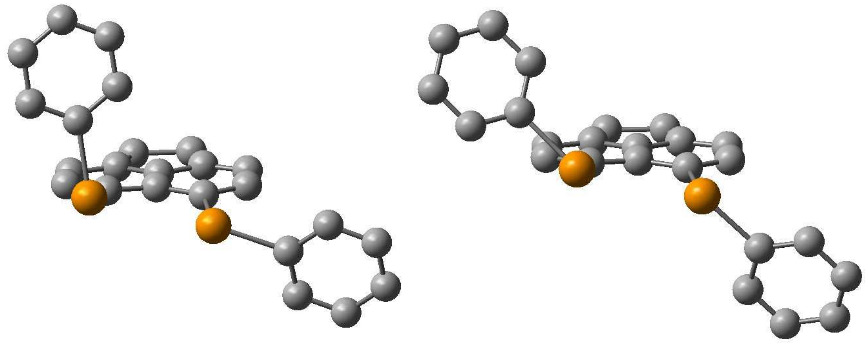

Figure S1: B3LYP-optimized conformations of **A1**: AB (left), CCt (right), Te-atoms orange, H-atoms omitted for clarity

Born-Oppenheimer molecular dynamics simulations were performed at the B3LYP/SDD/6-31G(d) level with a time step of 1 fs in an NVT ensemble at 320 K , assigning the mass of deuterium to all hydrogen atoms. The slightly elevated temperature was used to enhance conformational sampling. These simulations were carried out with the Chemshell program[12] using Turbomole as QM code (using a medium-sized grid (m3 keyword) and, to ensure proper energy conservation, tight SCF convergence criteria, keyword scfconv 8).

Indirect spin-spin coupling constants (SSCCs) were computed[13] at the BP86[8] and PBE0[7] levels using the relativistic zeroth-order regular approximation both in its scalar formulation (ZORA)[14] and with spin-orbit coupling (ZSO),[15] together with a TZ2P basis of Slater-type orbitals and a fine integration grid (Integration 6). Evolution of the computed SSCC along the BOMD trajectory is illustrated in Figure S2. Scalar ZORA results have been decomposed into contributions from natural localized MOs (NLMOs) from a natural bond orbital (NBO) analysis, as described in reference 16. Selected NLMO contributions are collected in Table S1. These calculations were performed with the ADF program.[17,18]

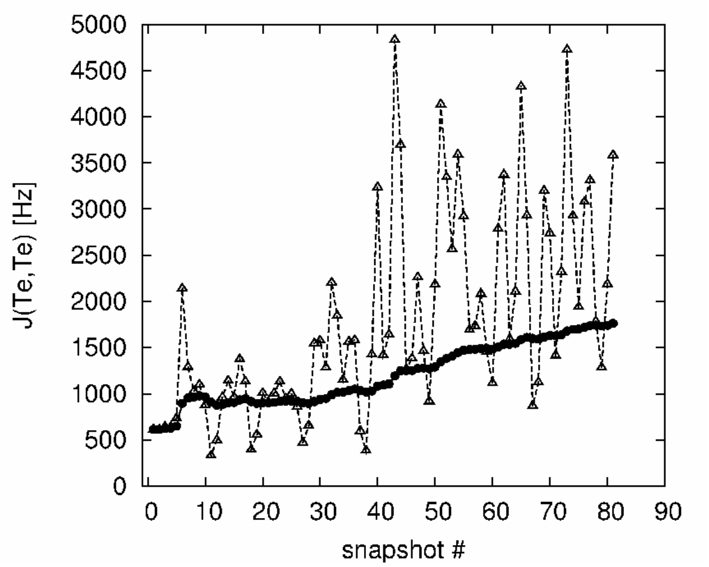

Figure S2: Evolution of the $J(^{125}Te,^{125}Te)$ SSCC in **N1a** along the BOMD/B3LYP trajectory (ZSO/BP level); triangles: instantaneous value, filled circles: running average. Note that the latter is not yet fully converged.

Table S1: Selected NLMO contributions to $K(^{125}Te,^{125}Te)$ [10^{19} kg m^{-2} C^{-2}] and $J(^{125}Te,^{125}Te)$ [Hz].[a]

NLMO no.	*K*	*J*
91,93 ("sp")[b]	821	995
92,94 ("p")[b]	341	413

[a]Scalar ZORA (BP86/TZ2P level); sum of local and nonlocal contributions; those from corresponding NLMOs on "perturbing" and "responding" nucleus have been averaged, as recommended in reference 16a. [b]Lone pairs on Te; these are the largest single contributions, the next largest stem from two core orbitals, at J = 181 Hz each, and from the Te-C(Me) σ-bonds, at J = -137 Hz each.

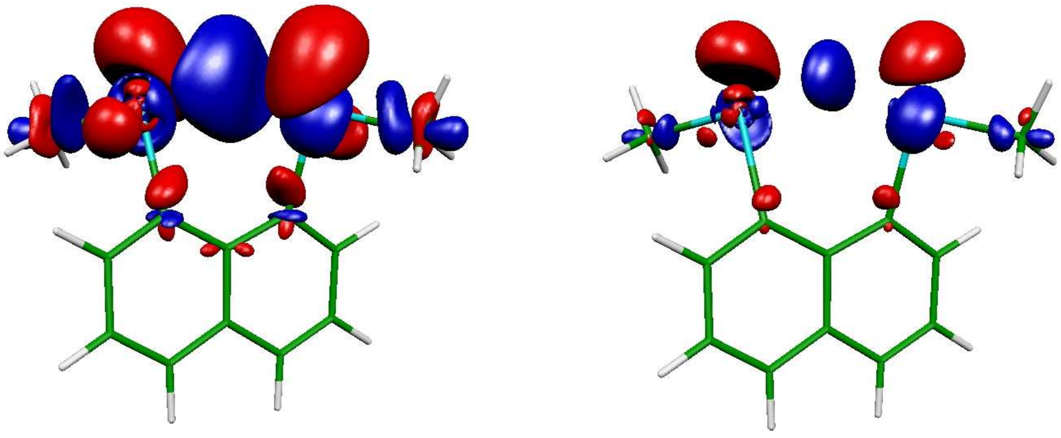

Figure S3: Relativistic scalar DKH-1[19] (left) and non-relativistic (right) CDD. Both isosurfaces plotted for CDD = 6.0.

Analysis of LMO contributions to the Fermi-contact part of $J(^{125}Te,^{125}Te)$ at the non-relativistic was done with a modified version of the deMon program[20] at the BP86 level and using the same TZVP/II basis set as employed in the nonrelativistic SSCC calculations. Coupling deformation density (CDD) calculations at the relativistic and non-relativistic levels were performed with a modified version of the ReSpect program[21] also at the BP86 level and using the Hirao basis set[22] on Te. Localized MOs (Figure S3) and the CCD have been visualized with the Molekel program.[23] Atomic calculations have been performed for P and Te at the BP86/TZVP/III level, affording 3s(P) and 5s(Te) densities of 6.5 a.u. and 22.4 a.u., respectively; because this is the main transmission mechanism for the FC part, and because the squared ratio of the gyromagnetic ratios of ^{31}P:^{125}Te is 1:1.627, $J(^{125}Te,^{125}Te)$ should be larger than $J(^{31}P,^{31}P)$ by a factor of $(22.4/6.5)^2/1.627 = 7.3$.

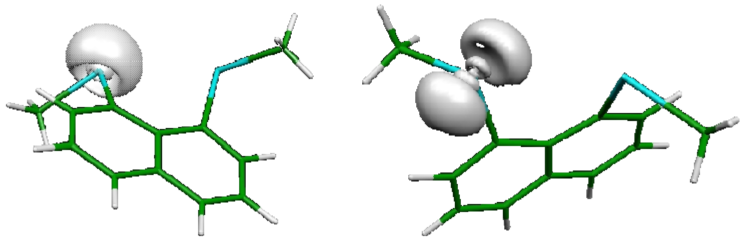

Figure S4: Densities $|\phi|^2$ of lone-pair orbitals on Te in **N1a**, obtained from Pipek-Mezey localization at the nonrelativistic BP86/TZVP/II level; left: "sp-type", right: "p-type".

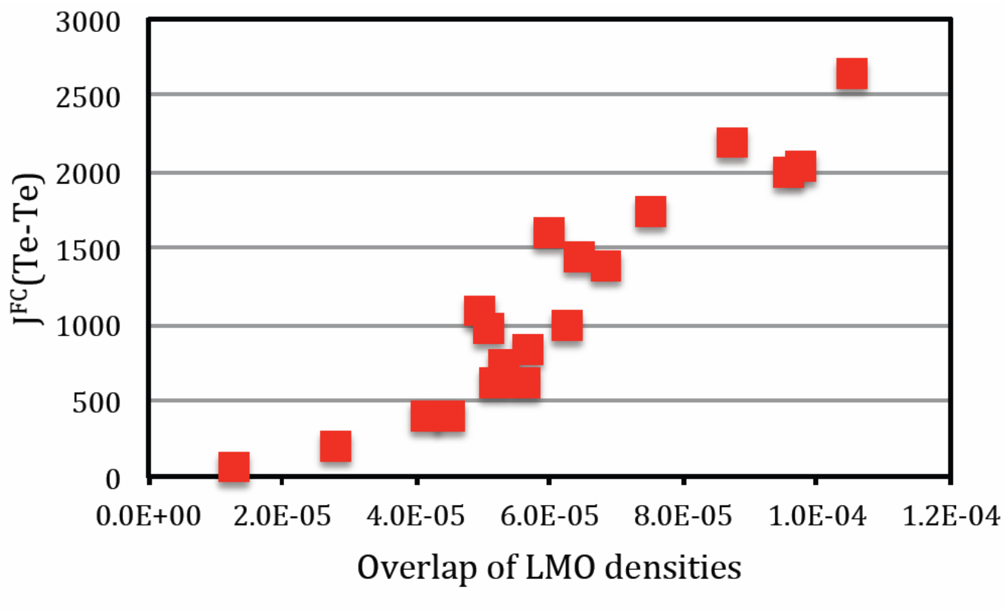

Figure S5: Plot of computed $J(^{125}Te,^{125}Te)$ values (in Hz, NR-BP86/TZVP/II level, FC part only) in **N1a** vs. the overlap of densities (in a.u.) of the unperturbed localized molecular orbitals representing the "sp"-like Te lone pairs (cf. Figure S3 left). Calculations were performed for the partially optimized structures from the 2D-scan in Figure 1 in the main paper.

Full Experimental Details

All experiments were carried out under an oxygen- and moisture-free nitrogen atmosphere using standard Schlenk techniques and glassware. Reagents were obtained from commercial sources and used as received. Dry solvents were collected from a MBraun solvent system. Elemental analyses were performed by Stephen Boyer at the London Metropolitan University. Infra-red spectra were recorded as KBr discs in the range 4000-300 cm^{-1} on a Perkin-Elmer System 2000 Fourier transform spectrometer. ^{1}H and ^{13}C NMR spectra were recorded on a Jeol GSX 270 MHz spectrometer with δ(H) and δ(C) referenced to external tetramethylsilane. ^{123}Te NMR and ^{125}Te NMR spectra were recorded on a Jeol GSX 270 MHz spectrometer with δ(Te) referenced to external diphenyl ditelluride respectively. Assignments of ^{13}C and ^{1}H NMR spectra were made with the help of H-H COSY and HSQC experiments. All measurements were performed at 25 °C. All values reported for NMR spectroscopy are in parts per million (ppm). Coupling constants (*J*) are given in Hertz (Hz). Mass spectrometry was performed by the University of St. Andrews Mass Spectrometry Service. Electrospray Mass Spectrometry (ESMS) was carried out on a Micromass LCT orthogonal accelerator time of flight mass spectrometer.

5,6-Bis(mesityltelluro)acenaphthene [A1a]: A solution of 5,6-dibromoacenaphthene (0.23 g, 0.96 mmol) in diethyl ether (40 mL) was cooled to -10-0 °C on an ice-ethanol bath and to this was added a solution of TMEDA (0.4 mL, 2.55 mmol). The mixture was allowed to stir for 15 min before a solution of *n*-butyllithium (2.5 M) in hexane (0.9 mL, 2.31 mmol) was added dropwise over a period of 15 min. During these operations, the temperature of the mixture was maintained at -10-0 °C. The mixture was stirred at this temperature for a further 1 h, before being cooled to -78 °C. A solution of dimesityl ditelluride (0.96 g, 1.92 mmol) in diethyl ether (100 mL) was then added dropwise and the resulting solution was stirred at -78 °C for a further 1 h. The mixture was allowed to warm to room temperature and then washed with 0.1 N sodium hydroxide (2 x 60 mL). The organic layer was dried over magnesium sulfate and concentrated under reduced pressure to afford a red solid. The crude product was washed with hexane affording a yellow crystalline solid which was collected by filtration. An analytically pure sample was obtained from recrystallisation by diffusion of hexane into a saturated solution of the compound in dichloromethane (0.14 g, 22%); mp 125-127 ºC (decomp); IR (KBr disk): ν_{max} cm^{-1}

3385w, 3018w, 2918s, 2855w, 2373w, 1889w, 1718s, 1699s, 1636w, 1594vs, 1565s, 1444vs, 1417s, 1377s, 1320s, 1292s, 1260w, 1209w, 1107w, 1029s, 949w, 922w, 843vs, 696vs, 612vs, 540w, 467s, 408w, 329w; ^{1}H NMR (270 MHz, $CDCl_3$, 25 °C, TMS) δ = 7.33 (2 H, d, $^3J_{HH}$ 7.3, Acenap 4,7-H), 6.80 (4 H, s, Te*Mes* 13,15,18,22-H), 6.75 (2 H, d, $^3J_{HH}$ 7.3, Acenap 3,8-H), 3.12 (4 H, s, 2 x CH_2), 2.30 (12 H, s, 4 x CH_3), 2.15 (6 H, s, 2 x CH_3); ^{13}C NMR (67.9 MHz; $CDCl_3$; 25ºC; Me_4Si) δ = 147.2(q), 144.9(q), 144.6(q), 141.0(q), 139.2(q), 138.8(s), 128.2(s), 126.9(q), 121.5(s), 114.5(q), 30.2(s, 4 x CH_3), 29.2(s, 2 x CH_3), 21.5(s, 2 x CH_2); ^{123}Te NMR (70.7 MHz, $CDCl_3$, 25 °C, PhTeTePh): δ = 362.9(s, J(^{123}Te-^{125}Te) 2818.5).

Crystal structure analysis

Data for **A1a** were determined at -148(1) °C on the St Andrews Robotic Diffractometer[24] a Rigaku ACTOR-SM, Saturn 724 CCD area detector with graphite monochromated Mo-Kα radiation (λ = 0.71073 Å). The data was corrected for Lorentz, polarisation and absorption. The data was collected and processed using CrystalClear (Rigaku).[25] The structures were solved by direct methods[26] and expanded using Fourier techniques.[27] The non-hydrogen atoms were refined anisotropically. Hydrogen atoms were refined using the riding model. All calculations were performed using the CrystalStructure[28] crystallographic software package except for refinement, which was performed using SHELXL-97.[29] These X-ray data can be obtained free of charge via www.ccdc.cam.ac.uk/conts/retrieving.html or from the Cambridge Crystallographic Data centre, 12 Union Road, Cambridge CB2 1EZ, UK; fax (+44) 1223-336-033; e-mail: deposit@ccdc.cam.ac.uk.

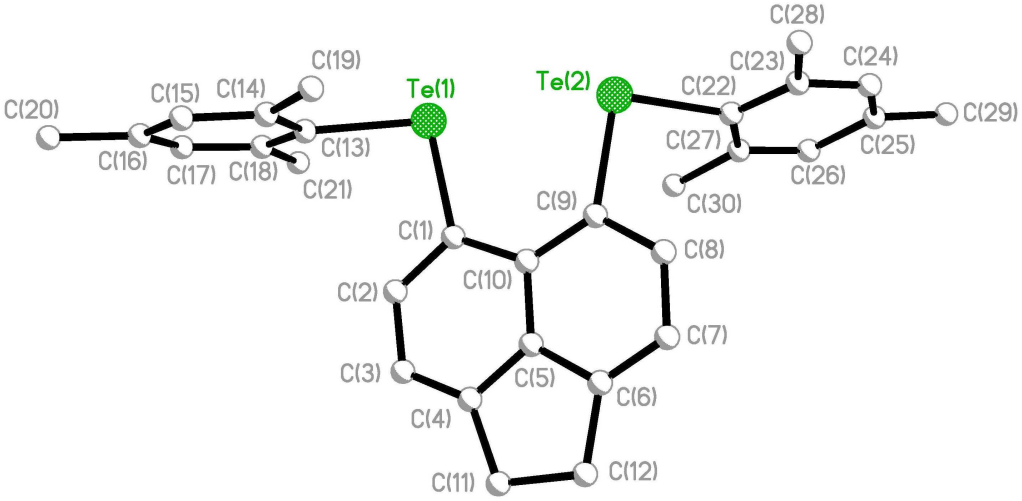

Figure S6. **The crystal structure of 5,6-bis(mesityltelluro)acenaphthene [Acenap(TeMes)$_2$]** (A1a**, H atoms omitted for clarity).**

Table S2. Crystallographic data for Acenap(TeMes)$_2$ (**A1a**).

	Acenap(TeMes)$_2$
Empirical Formula	$C_{30}H_{30}Te_2$
Formula Weight	645.77
Temperature (°C)	-148(1)
Crystal Colour, Habit	yellow, platelet
Crystal Dimensions (mm^3)	0.120 X 0.120 X 0.030
Crystal System	triclinic
Lattice Parameters	a = 7.668(2) Å
	b = 12.730(4) Å
	c = 13.289(4) Å
	α = 81.523(12)°
	β = 80.835(12)°
	γ = 84.403(13)°
Volume (Å^3)	V = 1263.0(6)
Space Group	P-1
Z value	2
Dcalc (g/cm^3)	1.698
F000	628
μ(MoKα) (cm^{-1})	23.256
No. of Reflections Measured	11117
Rint	0.0462
Min and Max Transmissions	0.760 - 0.933
Independ. Reflection (No. Variables)	5531(289)
Reflection/Parameter Ratio	19.14
Residuals: R_1 (I>2.00σ(I))	0.0617
Residuals: R (All reflections)	0.0839
Residuals: wR_2 (All reflections)	0.2221
Goodness of Fit Indicator	1.159
Flack Parameter	-
Maximum peak in Final Diff. Map	0.95 e^-/Å^3
Minimum peak in Final Diff. Map	-1.69 e^-/Å^3

Table S3. Selected interatomic distances [Å] and angles [°] for Acenap(TeMes)$_2$ (**A1a**, see Figure S6 for atom numbering).

**Parameter**	**Acenap(TeMes)$_2$**
	TeMes, TeMes
Peri-region-distances	
**X(1)···E(1)**	3.3380(11)
**Σr_{vdW} - X···E; % Σr_{vdW}[a]**	0.782; 81
**Te(1)-C(1)**	2.141(7)
**Te(2)-C(9)**	2.136(7)
Acenaphthene bond lengths	
**C(1)-C(2)**	1.390(10)
**C(2)-C(3)**	1.401(11)
**C(3)-C(4)**	1.357(12)
**C(4)-C(5)**	1.405(11)
**C(5)-C(10)**	1.429(10)
**C(5)-C(6)**	1.414(11)
**C(6)-C(7)**	1.355(11)
**C(7)-C(8)**	1.416(11)
**C(8)-C(9)**	1.406(12)
**C(9)-C(10)**	1.412(11)
**C(10)-C(1)**	1.429(11)
**C(4)-C(11)**	1.519(11)
**C(11)-C(12)**	1.536(12)
**C(12)-C(6)**	1.511(10)
Peri-region bond angles	
**Te(1)-C(1)-C(10)**	123.6(5)
**C(1)-C(10)-C(9)**	129.9(7)
**Te(2)-C(9)-C(10)**	126.5(6)
**Σ of bay angles**	**380.0(14)**
**Splay angle[b]**	20.0
**C(4)-C(5)-C(6)**	111.5(7)
**Te(2)-Te(1)-C(13)**	155.3(1)
**Te(1)-Te(2)-C(22)**	144.7(1)

Out-of-plane displacement		
**Te(1)**	-0.059(1)	
**Te(2)**	0.249(1)	
**C:(6)-(5)-(10)-(1)**	-177.1(7)	
**C:(4)-(5)-(10)-(9)**	179.5(7)	
Acenaphthene ring conformations		
**C(10)-C(1)-Te(1)-C(13)**	-152.1(6)	Acenap1: twist
**C(10)-C(9)-Te(2)-C(22)**	-136.4(6)	Acenap2: twist
Mesityl ring conformations		
**C(1)-Te(1)-C(13)-C(14)**	85.3(5)	Mes1: axial
**C(9)-Te(2)-C(22)-C(23)**	-107.2(5):	Mes2: axial

[a] van der Waals radii used for calculations: r_{vdW}(Te) 2.06Å, [b] Splay angle: Σ of the three bay region angles – 360.

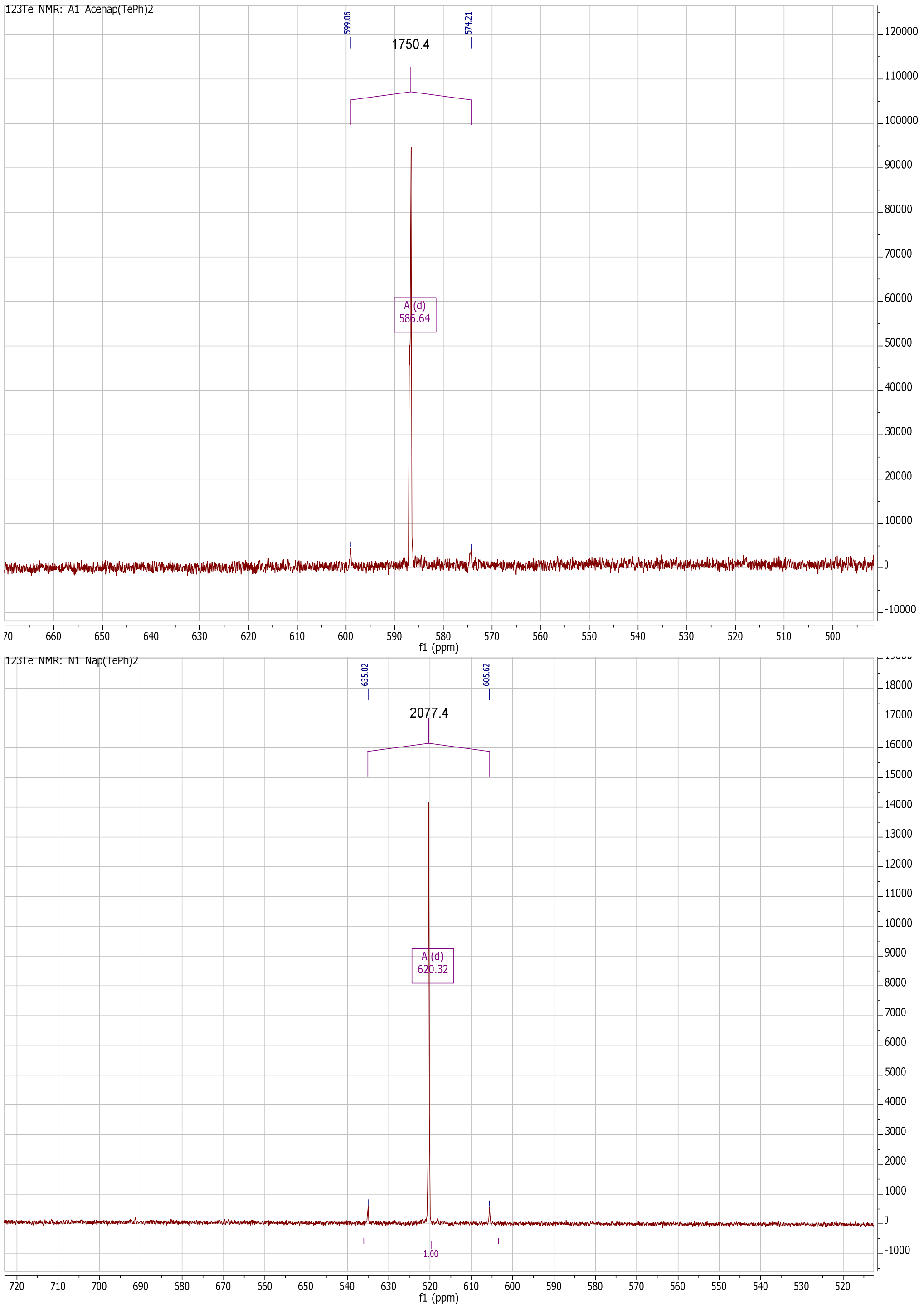

Figure S7: ^{123}Te NMR spectra for **A1** (top) and **N1** (bottom), showing the ^{125}Te satellites.

References

1 A. D. Becke, *J. Chem. Phys.,* 1993, **98**, 5648-5652; C. Lee, W. Yang and R. G. Parr, *Phys. Rev. B,* 1988, **37**, 785-789.

2 P. Schwerdtfeger, M. Dolg, W. H. E. Schwarz, G. A. Bowmaker and P. D. W. Boyd, *J. Chem. Phys.,* 1989, **91**, 1762-1774; A. Bergner, M. Dolg, W. Kuechle, H. Stoll and H. Preuss, *Mol. Phys.,* 1993, **80**, 1431-1441.

3 S. Huzinaga, J. Anzelm, M. Klobukowski, E. Radzio-Andzelm, Y. Sakai and H. Tatewaki, in: *Gaussian Basis Sets for Molecular Calculations*, Elsevier, Amsterdam, 1984.

4 K. B. Wiberg, *Tetrahedron*, 1968, **24**, 1083-1096.

5 A. E. Reed, F. Curtiss and L. A. F. Weinhold, *Chem. Rev.,* 1988, **88**, 899-926.

6 (a) F. R. Knight, A. L. Fuller, M. Bühl, A. M. Z. Slawin, J. D. Woollins, *Chem. Eur. J.* **2010**, *16*, 7605 -7616; (b) F. R. Knight, A. L. Fuller, M. Bühl, A. M. Z. Slawin, J. D. Woollins, *Inorg. Chem.* **2010**, *49*, 7577–7596; (c) L. K. Aschenbach, F. R. Knight, R. A. M. Randall, D. B. Cordes, A. Baggott, M. Bühl, A. M. Z. Slawin, J. D. Woollins, *Dalton Trans.* **2012**, *41*, 3141-3153; (d) F. R. Knight, K. S. A. Arachchige, R, A. M. Randall, M. Bühl, A. M. Z. Slawin, J. D. Woollins, *Dalton Trans.* **2012**, *41*, 3154-3165.

7 (a) J. P. Perdew, K. Burke, M. Ernzerhof, Phys. Rev. Lett. 1996, 77, 3865; (b) J. P. Perdew, K. Burke, M. Ernzerhof, Phys. Rev. Lett. 1997, 78, 1396; (c) C. Adamo, V. Barone, J. Chem. Phys. 1999, 110, 6158.

8 A. D. Becke, *Phys. Rev. A* **1988**, *38*, 3098-3100; J. P. Perdew, *Phys. Rev. B* **1986**, *33*, 8822; J. P. Perdew, *Phys. Rev. B* **1986**,*34*, 7406.

9 Turbomole Version 6.0, Turbomole GmbH, Karlsruhe, Germany (2009), cf: (a) R. Ahlrichs, M. Bär, M. Häser, H. Horn, H. Kölmel, Chem. Phys. Lett. 1989, 162, 165; (b) O. Treutler, R. Ahlrichs, J. Chem. Phys. 1995, 102, 346; (c) M. v. Arnim, R. Ahlrichs, J. Comput. Chem. 1998, 19, 1746.

10 W. Kutzelnigg, U. Fleischer, M. Schindler, in: *NMR Basic Principles and Progress,* Springer-Verlag: Berlin, **1990**; Vol. *23*, p 165.

11 (a) M. J. Frisch, G. W. Trucks, H. B. Schlegel, G. E. Scuseria, M. A. Robb, J. R. Cheeseman, J. A. Montgomery, Jr., T. Vreven, K. N. Kudin, J. C. Burant, J. M. Millam, S. S. Iyengar, J. Tomasi, V. Barone, B. Mennucci, M. Cossi, G. Scalmani, N. Rega, G. A. Petersson, H. Nakatsuji, M. Hada, M. Ehara, K. Toyota, R. Fukuda, J.Hasegawa, M. Ishida, T. Nakajima, Y. Honda, O. Kitao, H. Nakai, M. Klene, X. Li, J. E. Knox, H. P. Hratchian, J. B. Cross, V. Bakken, C. Adamo, J. Jaramillo, R. Gomperts, R. E. Stratmann, O. Yazyev, A. J. Austin, R. Cammi, C. Pomelli, J. W. Ochterski, P. Y. Ayala, K. Morokuma, G. A. Voth, P. Salvador, J. J. Dannenberg, V. G. Zakrzewski, S. Dapprich, A. D. Daniels, M. C. Strain, O. Farkas, D. K. Malick, A. D. Rabuck, K. Raghavachari, J. B. Foresman, J. V. Ortiz, Q. Cui, A. G. Baboul, S. Clifford, J. Cioslowski, B. B. Stefanov, G. Liu, A. Liashenko, P. Piskorz, I. Komaromi, R. L. Martin, D. J. Fox, T. Keith, M. A. Al-Laham, C. Y. Peng, A. Nanayakkara, M. Challacombe, P. M. W. Gill, B. Johnson, W. Chen, M. W. Wong, C. Gonzalez and J. A. Pople, Gaussian 03, Revision E.01, Gaussian, Inc., Wallingford CT, 2004; (b) M. J. Frisch, G. W. Trucks, H. B. Schlegel, G. E. Scuseria, M. A. Robb, J. R. Cheeseman, G. Scalmani, V. Barone, B. Mennucci, G. A. Petersson, H. Nakatsuji, M. Caricato, X. Li, H. P. Hratchian, A. F. Izmaylov, J. Bloino, G. Zheng, J. L. Sonnenberg, M. Hada, M. Ehara, K. Toyota, R. Fukuda, J. Hasegawa, M. Ishida, T. Nakajima, Y. Honda, O. Kitao, H. Nakai, T. Vreven, J. A. Montgomery, Jr., J. E. Peralta, F. Ogliaro, M. Bearpark, J. J. Heyd, E. Brothers, K. N. Kudin, V. N. Staroverov, R. Kobayashi, J. Normand, K. Raghavachari, A. Rendell, J. C. Burant, S. S. Iyengar, J. Tomasi, M. Cossi, N. Rega, J. M. Millam, M. Klene, J. E. Knox, J. B. Cross, V. Bakken, C. Adamo, J. Jaramillo, R. Gomperts, R. E. Stratmann, O. Yazyev, A. J. Austin, R. Cammi, C. Pomelli, J. W. Ochterski, R. L. Martin, K. Morokuma, V. G. Zakrzewski, G. A. Voth, P. Salvador, J. J. Dannenberg, S. Dapprich, A. D. Daniels, O. Farkas, J. B. Foresman, J. V. Ortiz, J. Cioslowski, and D. J. Fox, Gaussian 09, Revision A.02, Gaussian, Inc., Wallingford CT, 2009.

12 (a) ChemShell, a Computational Chemistry Shell, see www.chemshell.org (accessed May 2012); (b) P. Sherwood, A. H. de Vries, M. F. Guest, G. Schreckenbach, C. R. A. Catlow, S. A. French, A. A. Sokol, S. T. Bromley, W. Thiel, A. J. Turner, S. Billeter, F. Terstegen, S. Thiel, J. Kendrick, S. C. Rogers, J. Casci, M. Watson, F. King, E. Kerlson, M. Sjøvoll, A. Fahmi, A. Schäfer and C. Lennartz, *J. Mol. Struct. (THEOCHEM)* **2003**, *632*, 1-28.

13 (a) J. Autschbach, T. Ziegler, *J. Chem. Phys.* **2000**, *113*, 936; (b) J. Autschbach, *J. Chem. Phys.* **2008**, *129*, 094105.

14 (a) E. van Lenthe. E. J. Baerends, J. G. Snijders, *J. Chem. Phys.* **1994**, *101*, 9783; (b) E. van Lenthe, R. van Leeuwen, E. J. Baerends, J. G. Snijders, *Int. J. Quantum Chem.* **1996**, *57*, 281-293;(c) E. van Lenthe, E. J. Baerends, J. G. Snijders, *J. Chem. Phys*. **1993**, *99*, 4597.

15 E. van Lenthe, J. G. Snijders, E. J. Baerends, *J. Chem. Phys.* **1996**, *105*, 6505.

16 (a) J. Autschbach, *J. Phys. Chem.* **2007**, *127*, 124106; (b) J. Autschbach, *J. Chem. Phys*. **2008**, *128*, 164112

17 (a) E. J. Baerends, D. E. Ellis, P. Ros, *Chem. Phys.* **1973**, *2*, 41; (b) G. te Velde, E. J. Baerends, *J. Comput. Phys.* **1992**, *99*, 84; (c) G. te Velde, F. M. Bickelhaupt, E. J.Baerends, C. Fonseca Guerra, S. J. A. van Gisbergen, J. G. Snijders, T. Ziegler, *J. Comput. Chem*. **2001**, *22*, 931.

18 E.J. Baerends, T. Ziegler, J. Autschbach, D. Bashford, A. Bérces, F.M. Bickelhaupt, C. Bo, P.M. Boerrigter, L. Cavallo, D.P. Chong, L. Deng, R.M. Dickson, D.E. Ellis, M. van Faassen, L. Fan, T.H. Fischer, C. Fonseca Guerra, A. Ghysels, A. Giammona, S.J.A. van Gisbergen, A.W. Götz, J.A. Groeneveld, O.V. Gritsenko, M. Grüning, S. Gusarov, F.E. Harris, P. van den Hoek, C.R. Jacob, H. Jacobsen, L. Jensen, J.W. Kaminski, G. van Kessel, F. Kootstra, A. Kovalenko, M.V. Krykunov, E. van Lenthe, D.A. McCormack, A. Michalak, M. Mitoraj, J. Neugebauer, V.P. Nicu, L. Noodleman, V.P. Osinga, S. Patchkovskii, P.H.T. Philipsen, D. Post, C.C. Pye, W. Ravenek, J.I. Rodríguez, P. Ros, P.R.T. Schipper, G. Schreckenbach, J.S. Seldenthuis, M. Seth, J.G. Snijders, M. Solà, M. Swart, D. Swerhone, G. te Velde, P. Vernooijs, L. Versluis, L. Visscher, O. Visser, F. Wang, T.A. Wesolowski, E.M. van Wezenbeek, G. Wiesenekker, S.K. Wolff, T.K. Woo, A.L. Yakovlev, ADF2010.02, SCM, Theoretical Chemistry, Vrije Universiteit, Amsterdam, The Netherlands, http://www.scm.com (accessed May 2012).

19 R. Samzow, B.A. Hess, G. Jansen, *J. Chem. Phys*. **1992** *96* 1227.

20 D.R. Salahub, R. Foumier, P. Mlynarski, I. Papai, A. St-Amant and J. Ushio, in: Density Functional Methods in Chemistry, eds. J.K. Labanowski and J.W. Andzelm (Springer,Berlin, 1991) p. 77; modified version: V. G. Malkin, O. L. Malkina, A. Křístková, deMon-NMR program, version 2009.

21 V. G. Malkin, O. L. Malkina, R. Reviakine, A. V. Arbuznikov, M. Kaupp, B. Schimmelpfennig, I. Malkin Ondik, M. Repisky´ , S. Komorovsky´ , P. Hrobarik, E. Malkin, T. Helgaker and K. Ruud, ReSpect program version 2.2, 2012

22 T.Nakajima, K. Hirao. *J. Chem. Phys*. **2002**, *116* , 8270.

23 MOLEKEL 4.0, P. Flükiger, H. P. Lüthi, S. Portmann, J. Weber, Swiss Center for Scientific Computing, Manno (Switzerland), 2000.

24 Fuller, A. L.; Scott-Hayward, L. A. S.; Li, Y.; Bühl, M.; Slawin, A. M. Z.; Woollins, J. D. *J. Am. Chem. Soc.* **2010**, *132*, 5799–5802.

25 CrystalClear 1.6: Rigaku Corporation, 1999. CrystalClear Software User's Guide, Molecular Structure Corporation, (c) 2000. Flugrath, J. W. P. *Acta Crystallogr., Sect. D* **1999**, *D55*, 1718.

26 SIR97: Altomare, A.; Burla, M.; Camalli, M.; Cascarano, G.; Giacovazzo, C.; Guagliardi, A.; Moliterni, A.; Polidori, G.; Spagna, R. *J. Appl. Cryst*. **1999**, *32*, 115.

27 DIRDIF99: Beurskens, P. T.; Admiraal, G.; Beurskens, G.; Bosman, W. P.; de Gelder, R.; Israel, R.; Smits, J. M. M. 1999. The DIRDIF-99 program system, Technical Report of the Crystallography Laboratory, University of Nijmegen, The Netherlands.

28 CrystalStructure 3.8.1: Crystal Structure Analysis Package, Rigaku and Rigaku/MSC (2000-2006). 9009 New Trails Dr. The Woodlands TX 77381 USA.

29 SHELX97: Sheldrick, G.M. *Acta Crystallogr., Sect. A* **2008**, *64*, 112.
